# Supplementary material for: Analysing fast food consumption behaviour patterns: The case of Jordan
Source: PLoS One. 2025 Jun 27;20(6):e0326347. doi: 10.1371/journal.pone.0326347 (PMC12204562; doi:10.1371/journal.pone.0326347)
Supplement: Attachment — Submitted filename: Review_Fast Food Jordan.pdf [file pone.0326347.s003.pdf]

Dear Author,
Thank you for your valuable work. Below, I have provided some insights to help improve the paper:

1.
Methods Section:

I recommend including a graph that illustrates the key variable of interest, based on the theoretical framework, in the Methods section. This would enhance the readers' understanding of your approach and improve the overall readability of the paper.

I noticed that you defined the variable using the question format described in the paper cited at reference 20. I kindly invite you to elaborate on why a validated questionnaire was not utilized to investigate this area. Providing such insights would strengthen the clarity and robustness of your methodological approach.

2.
Figures 1 and 2: Consider redesigning Figures 1 and 2 to make them more visually appealing and clearer. A more polished design could increase their impact and effectiveness in conveying the intended message.

3.
Discussion Section: You stated, "Moreover, the study revealed a positive correlation between higher income, BMI, and frequent fast-food consumption." However, this finding contrasts with the existing literature, which generally suggests that higher BMI is associated with lower income. I suggest including references that establish this general trend, as well as studies specific to Jordan, to explore whether your findings reflect regional nuances.

4.
Income Variable: Since your study focuses on the student population, the use of "income" as a variable requires clarification. In this context, "income" likely refers to family income, which may not accurately represent students’ financial independence or spending habits. Furthermore, students often manage money that they do not directly earn, which limits the variable's relevance. I suggest reconsidering the role of income in your analysis or reframing its significance in your discussion.

5.
Economic Status and BMI: The expression, "This indicates that economic status and weight-

related factors significantly influence dietary choices," could be improved to reflect a more nuanced interpretation. Additionally, the sentence, "Addressing these economic barriers and providing affordable healthy food options could mitigate the impact of fast-food consumption, particularly among higher-income students," should consider broader social factors.

6.

Social Context of Fast-Food Consumption: Investigate whether fast-food consumption among students serves as a form of socialization or if students visit fast-food outlets alone. Understanding these social dynamics is crucial for devising strategies to reduce fast-food consumption. Merely emphasizing the health risks of fast food may not suffice to change behavior. Instead, addressing the social motivations behind fast-food visits could provide more effective interventions.

I hope these suggestions help improve your manuscript.
