# Supplementary material for: Analysing fast food consumption behaviour patterns: The case of Jordan
Source: PLoS One. 2025 Jun 27;20(6):e0326347. doi: 10.1371/journal.pone.0326347 (PMC12204562; doi:10.1371/journal.pone.0326347)
Supplement: Attachment — Submitted filename: Response to Reviewers.docx [file pone.0326347.s005.docx]

**Response to Reviewer Comments**

**Manuscript: Analysing Fast Food Consumption Behaviour Patterns: The Case of Jordan**

- Comment 1: Use of Non-Validated Questionnaire

We thank the reviewer for this important observation. While validated instruments offer consistency, we deliberately tailored our questionnaire to align with the behavioral, linguistic, and cultural context of Jordanian university students. The instrument retained the core structure of the Theory of Planned Behavior, adapting the extended theory constructs used by Dunn et al. (2011) to enhance relevance and clarity for the target population.

The adapted questionnaire was pilot-tested, revised based on feedback, and assessed for internal consistency (Cronbach’s alpha: 0.60–0.86). This approach is consistent with prior Theory of Planned Behavior-based research in non-Western settings.

To support this methodological framework, we have also added a graph in the Methods section (now Figure 1) that visually illustrates the study’s conceptual framework, based on the extended Theory of Planned Behavior, and key variables of interest. This addition improves reader comprehension of the theoretical structure guiding our analysis.

- Comment 2: Figures 1 and 2 - Clarity and Design

We appreciate the suggestion. Figures 1 and 2 have been redesigned and renamed as Figures 2 and 3, while a new Figure 1 was added in the Methods section to illustrate the full conceptual framework. The revised figures use clearer layoutsand improved labeling to enhance readability and impact.

- Comment 3: BMI- Income

Thank you for this insightful comment. We now reference Burgoine et al. (2018) to reflect global trends showing a negative relationship between income and BMI in high-income settings. To contextualize our contrasting result, we included Jordan-specific studies (Khader et al., 2009; Khatatbeh et al., 2022) showing that students with higher income tend to consume more fast food and exhibit higher BMI levels, likely due to greater access to calorie-dense options.

- Comment 4: Clarification of 'Income' Variable

We agree with the reviewer. The term “income” was clarified in the Methods section as referring to students’ available personal funds, including family support, stipends, part-time jobs, and scholarships. It serves as a proxy for financial flexibility rather than independence.

- Comment 5: Economic Status and BMI Interpretation

Thank you for this thoughtful comment. We agree that the original phrasing could be refined to better reflect the complexity of factors influencing dietary choices. In response, we have revised this section of the discussion to emphasize that dietary behavior is shaped by a multidimensional interplay of economic status, physiological characteristics (e.g., BMI), psychological dispositions, and social-environmental influences such as food marketing, peer behavior, and cultural norms. We also revised the second sentence to reflect that effective interventions should not only address affordability and access but also tackle social motivations and environmental cues that shape food choices among students. These revisions now provide a more comprehensive and context-sensitive interpretation of the findings.

- Comment 6: Social Context of Fast-Food Consumption

We fully agree. While our study did not explicitly assess whether students consumed fast food alone or in social groups, we agree that the social context of consumption is a critical factor in understanding food choices among young adults. In response, we have revised the Discussion section to acknowledge the likely social function of fast-food consumption, such as peer bonding, group outings, or convenience in communal settings. We also note that social motivations and norms may significantly influence dietary behavior, potentially limiting the effectiveness of health messaging alone. The revised text emphasizes the need for interventions that consider social dynamics, and we recommend that future research explore whether students eat alone or with peers, and how group behaviors shape consumption decisions.

**Additional comments:**

- Comment: Methods and Results - Clarity and Consistency

Thank you for the feedback. The Methods and Results sections have been revised for clarity and conciseness. To address the discrepancy, we clarified that 408 students participated and ensured consistency across the abstract and body of the paper. The phrasing "around 400" in the methodology reflects the initial planning stage; the final figure of 408 is used consistently in the Results and Abstract.

- Comment: Language and Typographical Errors

We appreciate this note. The entire manuscript has undergone a thorough language and grammar review. Typos and stylistic inconsistencies have been corrected to improve clarity and ensure professional quality.

- Comment: Sampling and Representativeness

We acknowledge this important point. In response, we have clarified in the Methods section that the University of Jordan was selected due to its size, diversity of academic programs, and geographic centrality. These features make it a suitable and meaningful cross-section for studying university students in Jordan. While the sample may not fully represent all students nationwide, its demographic diversity provides strong grounds for initial exploration. We also addressed the gender imbalance in the sample, noting that the high proportion of female participants (approximately 75%) aligns with national enrollment patterns in Jordanian universities, particularly within the humanities and social sciences. This clarification supports the interpretability and relevance of the findings within the context of the local higher education landscape.

- Comment: The Introduction section needs revision.

The Introduction has been thoroughly revised to include a clear research aim, theoretical assumptions, justification for the population focus, explicit objectives, and a statement of the study’s originality and contribution to the field.

**We thank the editor and reviewers for their valuable comments, which significantly improved the clarity and rigor of this paper.**
